# Developmental changes in lung function of mice are independent of sex as a biological variable

**DOI:** 10.1152/ajplung.00120.2023

**Published:** 2024-02-20

**Authors:** Thomas Bärnthaler, Abhay B. Ramachandra, Sadè Ebanks, Nicole Guerrera, Lokesh Sharma, Charles S. Dela Cruz, Jay D. Humphrey, Edward P. Manning

**Affiliations:** ^1^Division of Pharmacology, Otto Loewi Research Center, Medical University of Graz, Graz, Austria; ^2^Department of Biomedical Engineering,Yale University, New Haven, Connecticut, United States; ^3^Section of Pulmonary, Critical Care, and Sleep Medicine, Yale University, New Haven, Connecticut, United States; ^4^VA Connecticut Healthcare System, West Haven, Connecticut, United States; ^5^Department of Medicine (Cardiovascular Medicine), Yale Translational Research Imaging Center, Yale University, New Haven, Connecticut, United States

**Keywords:** allometry, development, lung function, sex as a biological variable

## Abstract

Pulmonary function testing (PFT) in mice includes biomechanical assessment of lung function relevant to physiology in health and its alteration in disease, hence, it is frequently used in preclinical modeling of human lung pathologies. Despite numerous reports of PFT in mice of various ages, there is a lack of reference data for developing mice collected using consistent methods. Therefore, we profiled PFTs in male and female C57BL/6J mice from 2 to 23 wk of age, providing reference values for age- and sex-dependent changes in mouse lung biomechanics during development and young adulthood. Although males and females have similar weights at birth, females weigh significantly less than males after 5 wk of age (*P* < 0.001) with largest weight gain observed between 3 and 8 wk in females and 3 and 13 wk in males, after which weight continued to increase more slowly up to 23 wk of age. Lung function parameters including static compliance and inspiratory capacity also increased rapidly between 3 and 8 wk in female and male mice, with male mice having significantly greater static compliance and inspiratory capacity than female mice (*P* < 0.001). Although these parameters appear higher in males at a given age, allometric scaling showed that static compliance and inspiratory compliance were comparable between the two sexes. This suggests that differences in measurements of lung function are likely body weight-based rather than sex-based. We expect these data to facilitate future lung disease research by filling a critical knowledge gap in our field.

**NEW & NOTEWORTHY** This study provides reference values for changes in mouse lung biomechanics from 2 to 23 wk of age. There are rapid developmental changes in lung structure and function of male and female mice between the ages of 3 and 8 wk. Male mice become noticeably heavier than female mice at or about 5 wk of age. We identified that differences in normal lung function measurements are likely weight-based, not sex-based.

## Graphical abstract

**Figure:**
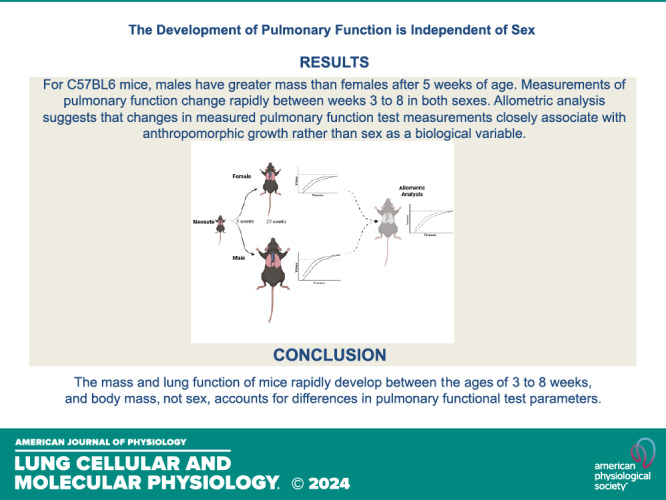

## INTRODUCTION

Mice are a commonly used model of human respiratory diseases (1, 2). Pulmonary function testing (PFT) in mice includes biomechanical measurements of lung function that are relevant to physiology in health and its alteration in disease, hence, it is frequently used in preclinical modeling of human lung pathologies (2, 3). Although multiple methods are being used to perform PFT in small animals, invasive forced maneuvers are believed to most reliably reflect pathology-associated changes (3, 4). These tests have been applied successfully to measure altered lung function in animal models of asthma, pulmonary fibrosis, emphysema, and acute lung injury, among other lung pathologies (4). Use of PFT is crucial to link biochemical changes with functional improvement in the lung as seen in recent discoveries of new therapeutic agents, such as nintedanib and pirfenidone in the treatment of idiopathic pulmonary fibrosis (5, 6).

**Figure 1.**
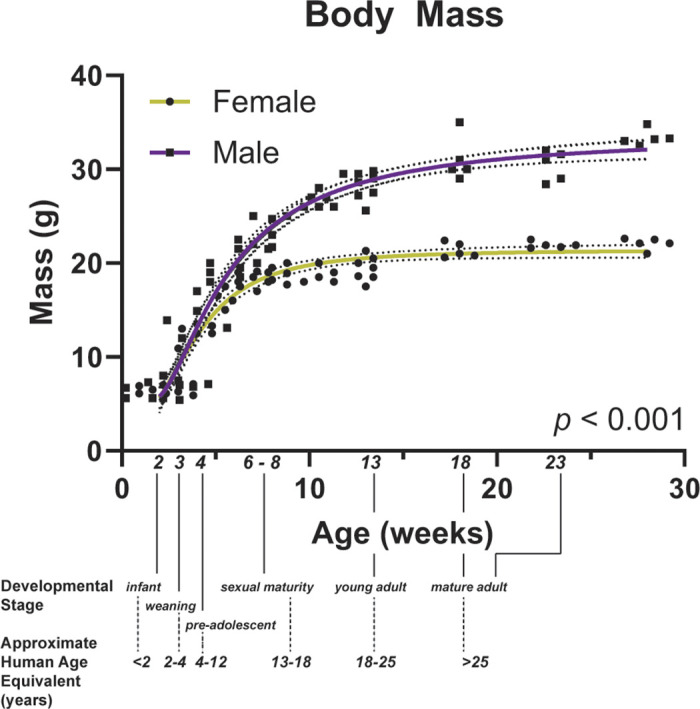
Changes in murine body mass over time correspond to human developmental milestones and are reflected by a monotonic curve. Body mass of male and female mice from 2 to 23 wk old. Developmental milestones of mice and near equivalent range of ages of humans are superimposed on the *x*-axis.

Of note, mice of different ages can be used to model different human pathologies. For example, broncho-pulmonary dysplasia studies utilize mice 2–3 wk of age. Other popular models of lung pathologies, such as the bleomycin model of pulmonary fibrosis, are commonly carried out in mice that are 10–12 wk of age or older whereas the ovalbumin-induced model of asthma is usually performed in mice 6–8 wk of age. On the other hand, models of chronic obstructive pulmonary disease (COPD) often take months of cigarette smoking thus necessitating mice of >20 wk of age at the time of lung function measurements (7). Importantly, these murine ages correspond loosely to diverse human age groups (see Fig. 1) ranging from preadolescence to adults to naturally aged with related ramifications.

Despite age being an important factor in mouse and human PFTs, data on age-associated changes in physiological lung function are scarce in mice. One reason for this could be the relative novelty of systems that conveniently and accurately measure these parameters. Although the first study to examine age-associated changes in static compliance in mice dates back to 1979 (8), commercial devices for mouse PFT measurement have only been widely marketed since 1997 and remain costly (9). Another reason for the scarcity of consistent age-associated changes in PFTs is the diversity of mouse strains used for experiments as well as the different choices of ages associated with PFT measurements. For example, static compliance was measured in 1–28-mo-old BALBc mice ∼40 years ago (8), but it was only recently that functional residual capacity, elastance, and methacholine responsiveness were measured in younger (2–8 wk of age) BALBc mice (10). Pressure-volume (*P-V*) relationships and compliance were not measured in these recent studies, and evaluations beyond 8 wk of age were not reported. In addition, sex differences in the physiological lung function have received little attention in these measurements.

Over the past decade, investigations of sex as a biological variable in diverse human diseases and their treatment have appropriately increased. This development resulted from the recognition that women’s health was often neglected in both preclinical and clinical studies, which is associated with delayed diagnosis, more adverse reactions to drugs (11), and worse outcomes (12). Emerging efforts to understand sex-specific differences in symptoms, risk factors, and effective drug dosing have yielded some beneficial effects on women’s health as seen in assessments and treatment of heart failure (13). In pulmonary medicine, anatomical and physiological differences between females and males are numerous in the respiratory tract and associated respiratory mechanics, and have recently been reviewed (14). Despite these well-known differences, there are limited data on effects of sex on baseline PFT parameters in mice, especially in invasive forced maneuvers, though biological sex is known to influence responses to many stimuli such as lipopolysaccharide (15), methacholine (16), and cigarette smoke exposure (17). Existing studies comparing baseline measurements between female and male mice have been conducted in adults, between 10 and 14 wk of age, with a focus on variability across mouse strains (18, 19).

There is, therefore, a critical knowledge gap in age- and sex-associated pulmonary biomechanics in mice, particularly as they develop from neonates to adults, which includes periods when mice are commonly used as models of human physiology and disease. In this study, we report results from PFTs in female and male C57BL/6J mice from 2 to 23 wk of age to determine age- and possible sex-based differences in pulmonary mechanics. This study thus provides reference values to which future mouse studies can be compared, enabling researchers to better plan murine studies to include age and sex and thereby increase rigor and reproducibility.

## METHODS

### Mouse Models

All experimental procedures were approved by the Yale Institutional Animal Care and Use Committee. Healthy female and male C57BL/6J mice from 2 to 23 wk of age were used to quantify pulmonary function. Although difficult to map mouse age to the corresponding human age precisely, we selected the following ages to reflect distinct human developmental stages and milestones (20, 21):

- Early postnatal development (mouse 2 wk old; human <2 yr old)
- Weaning (mouse 3 wk old; human 2–4 yr old)
- Preadolescence (mouse 4–6 wk old; human 4–12 yr old)
- Puberty and earliest reproducing age (mouse 6–8 wk old; human 13–18 yr old)
- Young adulthood (mouse 10–13 wk old; human 18–25 yr old)
- Mature adult (mouse 18 wk old; human 25–30 yr old)
- Middle age adult (mouse 23 wk old; human 30–40 yr old)

These ages and milestones are included in Fig. 1. All mice were weighed prior to the start of experiments.

### Micro-Computed Tomography and Morphometric Body Measurements

Three male and three female mice that were 3, 4, 5.5, 7, 8, 10.5, 13, and 18 wk old were scanned in a supine position using an in vivo small animal hybrid SPECT/high-resolution computed tomography (CT) scanner (MI Labs USPECT4/CT). Mice were sedated with 1.5% isoflurane inhalation via nose cone. Thoracic movement was detected by a motion sensor adhered to the mouse’s thorax allowing division of the respiratory cycle into four phases ranging from maximal inspiration to maximal expiration. Images were obtained throughout the respiratory cycle and sorted to the appropriate phase of respiration during postacquisition processing. Three-dimensional (3-D) images were constructed using 3-D Slicer 4.10.2 r28257 (22) in which we segmented aerated regions of lung using Houndsfield units (HU) for air as the threshold. The threshold for this study was set to −350 HU. Thoracic lung diameter was measured in micro-CT (mCT) images at the level of the heart using the external surface of the ribs and external intercostal muscles. The tail of each mouse was marked and the thoracic diameter, body mass, length of each mouse were recorded to correspond with their respective mCT measurement. We used Vernier calipers to measure thoracic diameter estimating the level of the heart at which to make the measurement. We standardized the measurement of body length for the mice by measuring the tip of the snout to its anus (TOSTA). The mice were then recovered.

### Lung Function

Gross lung function was quantified using the FlexiVent apparatus (SCIREQ, Montreal, QC, Canada) and established methods (23, 24). Specifically, mice were anesthetized with urethane (intraperitoneal injection, 1.5–2.0 g/kg), and the depth of anesthesia was assessed as the lack of response to a toe pinch, with supplemental injections given as needed. Once adequately anesthetized, the mouse was placed supine and the trachea was cannulated with a 20-gauge tracheostomy tube. The tube was inserted through a small ventral incision made in the rostral-most part of the trachea and advanced 3 mm caudal to the incision. The tube was then held securely in place with a suture tied around the trachea. The chest remained closed during all measurements. Mice were mechanically ventilated using the SCIREQ FlexiVent apparatus with 150 breaths/min, a tidal volume of 10 mL/kg body mass, and a positive end-expiratory pressure of 3 cmH_2_O prior to lung function measurements. Mice were then paralyzed with an intraperitoneal injection of pancuronium bromide (1 mg/kg). With the maximal vital capacity perturbation (called total lung capacity by SCIREQ), the inspiratory capacity of the lungs was determined using the SCIREQ software (Flexiware v.7.6, Service Pack 6). Forced oscillation perturbations (“quickprime-3”) subsequently measured tissue damping, reflecting energy dissipation within the lung parenchyma. Pressure-volume loops were calculated through quasi-static stepwise pressure-guided measurements of pressure *P* and volume *V*. SCIREQ software calculated static compliance by fitting the Salazar–Knowles equation (25)

$$V=\left(A-Be^{-kP}\right)$$

to the expiratory portion of the loop (26–28) and hysteresis, the area between inspiratory and expiratory loops, representing energy lost from the respiratory system between inspiration and expiration (29, 30). All maneuvers and perturbations were performed until three consistent measurements were achieved per mouse. A coefficient of determination of 0.9 was the lower limit for accepting a measurement.

### Lung Morphometric Assessment

Following pulmonary function measurement, mice were euthanized for lung inflation and harvest. Briefly, lungs were flushed with cold PBS and distended with low-melting agarose (0.5% in PBS) delivered via a tracheal tube under a constant pressure at 20 cmH_2_O. Once the agarose ceased to flow, an additional 5 min were allowed for the agarose to distribute within the lung. The lungs were then explanted and fixed overnight in 10% neutral-buffered formalin. Finally, three 5-µm thick lung sections were obtained from paraffin-embedded tissue blocks of at least three mice per age group, stained with hematoxylin and eosin, and digitized images were acquired with a Nikon DS-Ri2 bright-field microscope. A minimum of six random fields were evaluated by microscopic projection and the NIH ImageJ software. We avoided areas of large airways or blood vessels when possible. Alveolar size was estimated by semiautomated air space mean chord length measurement of ×20 hematoxylin and eosin (H&E) images using the macro developed by Crowley et al. (31).

### Allometric Scaling

Given the lower body mass of female mice at all ages beyond 5 wk, we considered possible allometric scaling of the form:

$$Y=kM^{\alpha }$$

where *Y* is a metric of lung function, *M* is the body mass (in grams), and α and *k* are the allometric constants determined from linear regression of data from male mice plotted as:

$$\log\left(Y\right)=\log\left(k\right)+\alpha \log\left(M\right)$$

A short description of allometric analysis is included in Supplemental Material. This enables proper interpretations of measured lung function parameters and thus determines weight-dependent versus sex-dependent changes in the lung function (26, 32–34).

### Statistics

Statistical analysis comparing female and male PFT measurements was accomplished using GraphPad Prism version 9.0. We used an *F* test to compare nonlinear fits for each set of data. Differences were considered significant at *P* < 0.05. Data in Volume-Pressure loops and morphometric analyses are represented as means ± standard deviation. Replicates of compliance, inspiratory capacity (IC), hysteresis, and tissue damping are shown. Allometric analysis was accomplished using MatLab 2019a.

## RESULTS

### Body Mass as a Function of Age

Body weight (mass times gravity) increases with the age, with the growth spurt during adolescent years correlating with the period of greatest anthropomorphic growth in humans. Changes in body mass with age are shown in Fig. 1 with superimposed age-related milestones in mice and humans. Body mass increased 3.4-fold in females and 5.6-fold in males from 2 wk of age to 23 wk of age. The largest increases were observed between 3 and 8 wk of age in females and 3 and 13 wk in males (Fig. 1), after which weight continued to increase more slowly up to 23 wk old. Although females and males have similar weights at birth, females weigh less than males after 5 wk of age. A table of mass and other data from this study is included for reference in Table 1.

**Table 1.** Reference values of data obtained from this study

	Female											Male									
Age, wk	2	3	4	5.5	7	8	10.5	13	18	23		2	3	4	5.5	7	8	10.5	13	18	23
Body mass, g																					
Mean	6.30	7.42	12.96	16.50	18.50	18.63	18.70	19.41	21.36	21.92		6.18	7.10	14.18	19.00	22.50	22.88	26.67	28.34	31.00	30.40
SD	0.40	2.02	0.46	0.95	0.50	0.84	0.84	1.31	0.79	0.35		0.72	0.90	1.90	0.79	1.46	2.09	0.82	1.46	2.35	1.61
Sample size, *n*	5	5	5	5	5	7	5	7	5	5		6	5	5	5	6	5	6	8	5	5
Static compliance, mL/cmH_2_O																					
Mean	0.009	0.010	0.031	0.037	0.042	0.050	0.058	0.067	0.064	0.070		0.012	0.017	0.034	0.043	0.057	0.055	0.052	0.075	0.078	0.082
SD	0.002	0.003	0.005	0.007	0.004	0.006	0.014	0.006	0.005	0.005		0.001	0.004	0.003	0.004	0.002	0.003	0.007	0.003	0.009	0.003
Sample size, *n*	5	5	5	3	5	5	3	5	5	5		3	5	5	3	3	5	4	5	5	5
Inspiratory capacity, mL																					
Mean	0.11	0.18	0.35	0.47	0.50	0.55	0.74	0.76	0.73	0.78		0.17	0.21	0.40	0.50	0.65	0.66	0.65	0.85	0.89	0.91
SD	0.03	0.06	0.05	0.09	0.04	0.08	0.26	0.05	0.04	0.06		0.04	0.05	0.04	0.04	0.04	0.03	0.06	0.02	0.08	0.04
Sample size, *n*	5	5	5	3	5	5	3	5	5	5		3	5	5	3	3	5	4	5	5	5
Hysteresis, mL·cmH_2_O																					
Mean	0.40	0.69	0.95	1.13	1.40	1.44	1.40	2.31	1.79	2.03		0.38	0.53	1.22	1.19	1.78	1.88	1.63	2.47	3.06	2.77
SD	0.23	0.42	0.31	0.19	0.24	0.41	0.18	0.33	0.14	0.25		0.02	0.16	0.35	0.18	0.60	0.49	0.53	0.61	0.71	0.45
Sample size, *n*	5	5	5	3	5	5	3	5	5	5		3	5	5	3	3	5	4	5	5	5
Tissue damping, cmH_2_O/mL																					
Mean	23.86	23.73	13.73	6.43	6.48	6.30	5.97	4.89	4.72	3.88		19.36	18.35	8.06	8.02	6.43	5.19	5.68	4.35	4.48	3.15
SD	4.87	8.67	2.20	1.11	1.61	1.11	1.31	0.60	0.31	0.23		4.04	4.19	0.87	1.51	1.13	1.00	0.77	1.18	1.79	0.31
Sample size, *n*	5	5	5	3	5	5	3	5	5	5		3	5	5	3	3	5	4	5	5	5
Length (TOSTA), mm																					
Mean		61.22	68.87	80.12	81.69	74.51	84.70	83.82	85.53				63.13	79.09	83.21	83.63	89.10	89.35	92.85	95.62	
SD		1.43	2.02	3.92	2.29	6.45	1.35	2.55	3.79				3.58	6.08	3.64	2.26	2.34	1.86	2.78	2.48	
Sample size, *n*		3	3	3	3	3	3	3	3				3	3	3	3	3	3	3	3	
Diameter (mCT), mm																					
Mean		14.92	16.41	16.50	16.05	16.75	17.55	15.84	17.25				14.03	16.57	18.19	16.96	18.78	18.08	17.63	20.47	
SD		0.60	2.10	1.96	1.22	1.85	0.58	2.13	1.43				2.03	1.39	1.72	0.23	1.27	0.62	2.65	2.86	
Sample size		3	3	3	3	3	3	3	3				3	3	3	3	3	3	3	3	

Sample size *n* refers to biological replicates or number of mice per experiment. IC, inspiratory capacity; mCT, micro-computed tomography; TOSTA, length from tip of snout to anus.

### Lung Function Changes with Age and Sex

**Figure 2.**
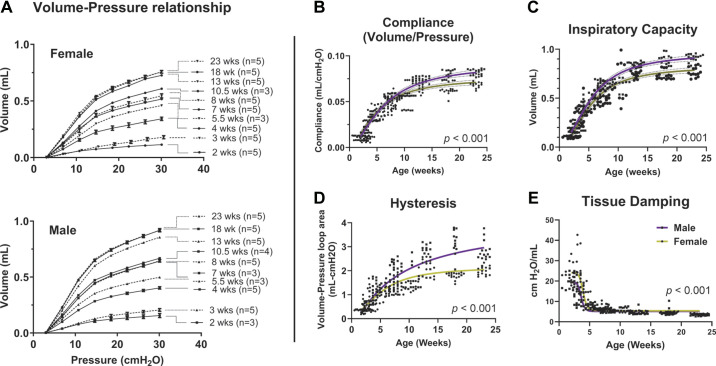
Changes in mechanical lung function are most pronounced between 2 and 13 wk of age. Mice were anesthetized and subjected to pulmonary function testing. Volume-pressure curves (*A*), static compliance (*B*), inspiratory capacity (*C*), hysteresis (*D*), and tissue damping (*E*) were determined using a FlexiVent device. *B*–*E*: a curve fit (purple for male, yellow for female) was performed to represent changes in lung function over time. Measurements were performed three times (technical replicates) for each mouse, and each replicate plotted.

In humans, the functional capacity of the lung increases until early adulthood, peaking around 20 (female) and 25 (male) years of age (35). In mice, lung pressure-volume (*P-V*) relationships (Fig. 2*A*) show a steep increase in dynamic lung compliance until 13 wk of age, after which the *P-V* relation does not change appreciably through 23 wk of age, suggesting a peak in lung function at this stage. When comparing *P-V* curves between males and females, there is no difference in dynamic compliance at 2 wk of age, but higher compliance in females at 3 wk of age. After 3 wk of age, dynamic compliance rose more rapidly in males and remained significantly higher at each corresponding age.

We also observed similar age- and sex-based trends in static compliance as measured during an inspiratory pause (Fig. 2*B*). Lung compliance increased with age in both sexes, with the steepest increases between weeks 3 and 13 when it plateaued. Overall, males had higher lung compliance at all ages.

Similar to lung compliance, total inspiratory capacity (IC) also increased with age (Fig. 2*C*). Compared with 2-wk-old mice, IC at 23 wk of age increased 7.5-fold in females and 5.6-fold in males. The most significant increases in IC occurred between the ages of 3 wk (weaning) and 13 wk (young adult stage) in both sexes, with a plateau in the adult stages. IC was higher in males at any age compared with females and continued to rise until 18 wk versus a plateau at 13 wk in females. Lung hysteresis also increased with age but peaked at 13 wk of age in females and 18 wk of age in males (Fig. 2*D*). The magnitude and rate of increase in hysteresis were significantly higher in males than in females. Tissue damping (Fig. 2*E*), a measure of parenchymal resistance, was highest at 2 and 3 wk of age but by adulthood decreased to less than 25% of its value during the preweaning ages. Damping was higher in females than in males at 2 and 3 wk of age but thereafter did not significantly differ between them.

### Structural Development of the Lung with Age as a Function of Sex

**Figure 3.**
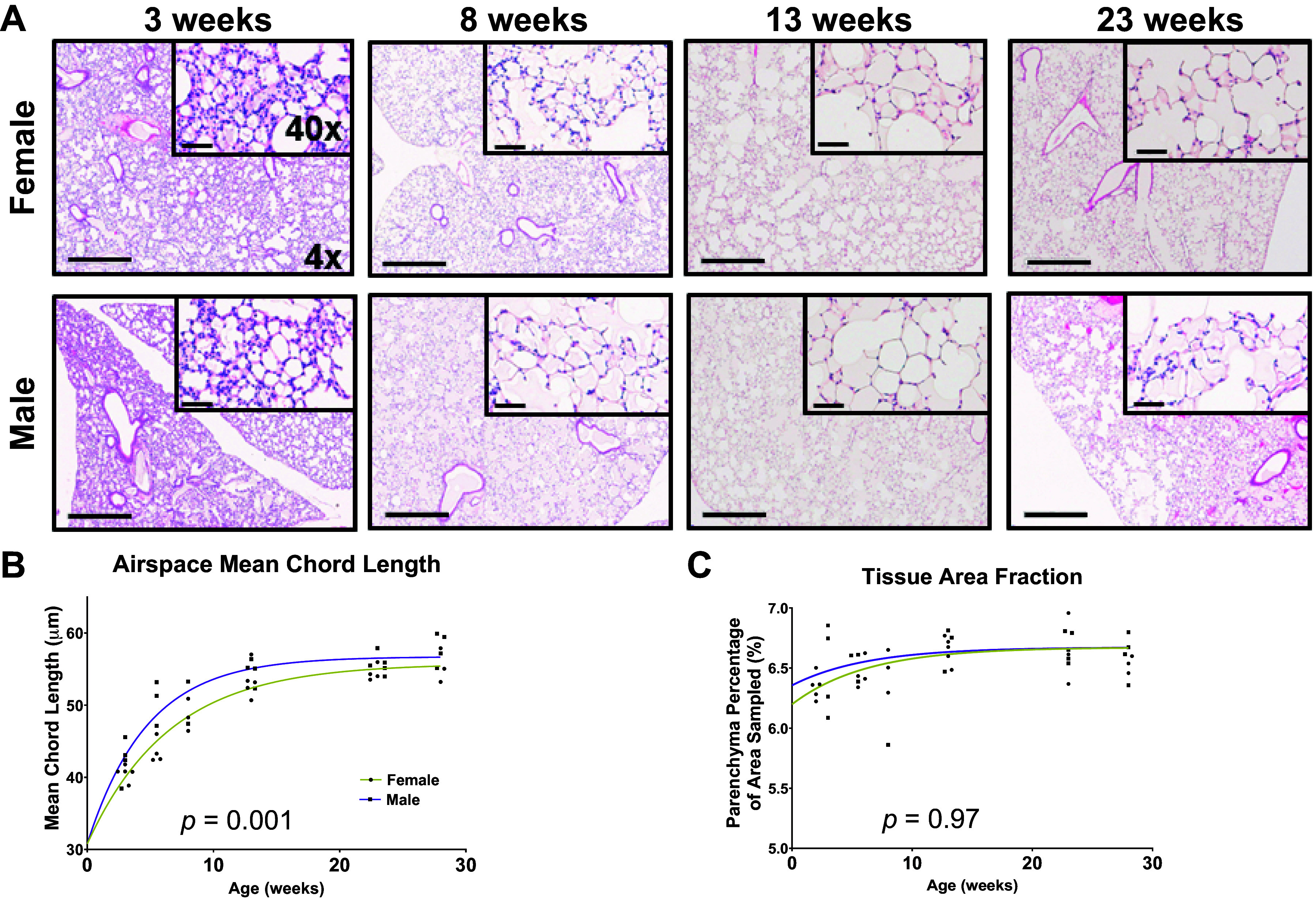
Mean chord length is higher in male mice and increases over time. *A*: representative histologic slides of lung slices stained with hematoxylin and eosin (H&E), ×4 with ×40 inset. Airspace mean chord length (*B*) and tissue area fraction (*C*) were derived from photomicrographs using ImageJ. Scale bars are 600 microns in ×4 and 60 microns in ×40.

To demonstrate that changes in structure that we observed within the lungs of mice are similar to results of others, we performed cursory morphometric analyses and compared them with previously reported structural data from mice of similar ages and sex (36). The lungs had prominent alveolarization at the youngest age studied, 3 wk (Fig. 3). Lung architecture continued to develop up to 13 wk of age, after which few changes were visible histologically. Alveolar size, as measured by mean chord length, gradually increased until 13 wk and was stable thereafter (Fig. 3*B*). Similarly, the tissue area fraction, a measure of parenchymal tissue, increased with age until stabilizing by 13 wk in both male and female mice (Fig. 3*C*). The curves for males and females are not significantly different for tissue area fraction. These changes are similar to structural changes previously described by others (Supplemental Fig. S2) (36).

### Allometric Scaling Demonstrates Similarities in Lung Function Parameters between Male and Female Mice

**Figure 4.**
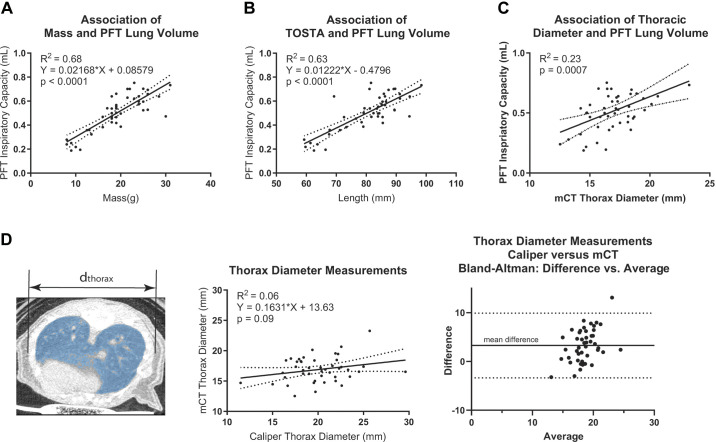
Association of body mass, length, and thoracic diameter with lung volume measured by pulmonary function testing. Lung volume correlates (in descending *R*^2^ order) with mass (*A*), length (*B*), and micro-computed tomography (mCT)-based thoracic diameter (*C*). *D*: a representative mCT image with respective diameter. Correlation and Bland–Altmann plot of mCT- and Vernier caliper-based measurements of thoracic diameter were performed.

To associate body mass, body length, and thoracic diameter of mice with lung volumes, we compared these three metrics [with body length standardized by measuring from the tip of the snout to its anus (TOSTA)] with their respective lung volumes in a subset of male and female mice ranging from 3 to 18 wk of age. Whereas height (body length) is a standard for estimating ideal lung volume in humans, in mice we found that differences in lung volumes of mice were explained by body mass (*R*^2^ = 0.68) as well or better than by length (*R*^2^ = 0.63) and better than thoracic diameter (*R*^2^ = 0.23, Fig. 4, *A–C*). The poor correlation between thoracic diameter and PFT lung volume is likely due to a high degree of inaccuracy of measuring thoracic diameter with calipers, as was confirmed by comparing measurements of thoracic diameter as might be done experimentally when performing PFTs with mCT, which yield accurate in situ measurements of the thoracic diameter (Fig. 4*D*). There is poor association between the two measurements of thoracic diameter (*R*^2^ = 0.06, Fig. 4*D*). The mean difference between caliper and mCT measurements of the thoracic diameters is 3.3 mm with a 95% limit of agreement of the means ranging from −3.4 to 9.9 mm, which reflects a variation that is exceedingly large for physiologic experiments.

**Figure 5.**
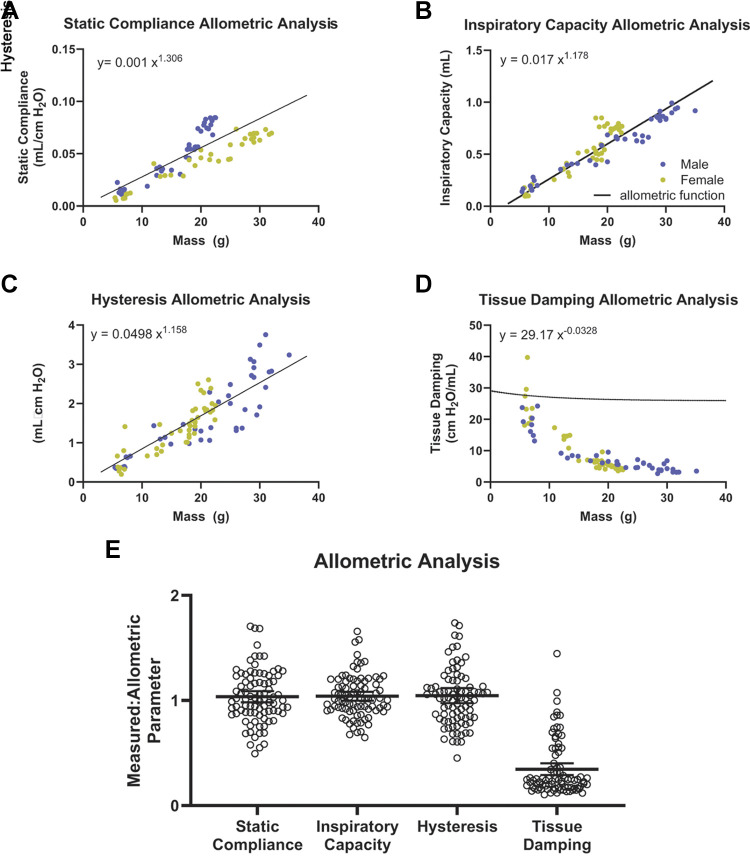
Body mass, not sex, accounts for differences in pulmonary functional test parameters. Allometric analysis was performed by comparing body mass to static compliance (*A*), inspiratory capacity (*B*), hysteresis (*C*), and tissue dampening (*D*). *E*: ratios for the respective parameters are given. Note that *x* denotes body mass M in panels *A–D*.

A basic statistical analysis suggests significant differences in lung function between males and females. Yet, it was possible that these differences resulted from different body sizes affecting the overall interpretation. To test for this possible confounder, we performed allometric scaling to determine body mass-dependent versus sex-dependent changes in measured lung function parameters. There is little difference in female compared with male lung function for most measurements after normalizing with respect to body mass (Fig. 5, *A–D*; Supplemental Fig. S1). One exception is tissue damping data; while male and female tissue damping data appear to behave similarly, our analysis suggests that body mass is not a good predictor of tissue damping in mice. The reasons for this remain unclear. The ratio of individual lung function measurements to body mass-normalized values, using relationships determined by allometric regression analysis, were plotted for each lung function parameter (Fig. 5*E*). The average value of the ratio for each lung function parameter was ∼1, suggesting that observed differences in lung function measurements seen in individual mice of different ages and sex are closely related to their respective body mass rather than sex as biological variables. Therefore, although all parameters appear higher in males at a given age, allometric scaling showed that IC, static compliance, and dynamic compliance are comparable between the two sexes.

## DISCUSSION

This study evaluated lung function using common forced invasive maneuvers in mice spanning the preweaning juvenile stage (2 wk of age) through adulthood (23 wk of age). The greatest rate of change of lung function parameters in this cohort occurred between 3 and 8 wk of age, corresponding to the period of greatest anthropomorphic growth. Importantly, allometric scaling revealed that measured age-dependent functional parameters of the lung tracked body mass, hence excluding sex-dependent differences. Furthermore, our histologic and morphometric evaluations revealed that alveolar size and proportion of tissue to air space also increased during the period of greatest change in lung function and plateaued after 13 wk. We submit that these age-related trends and sex-based differences in pulmonary function can serve as normal reference values for future studies in C57BL/6J mice as well as those having other backgrounds or conditions. More importantly, by applying these data to commonly used models of lung disease, there is the potential to better tailor therapy to developmental stages. To the best of our knowledge, this is the first report of developmental changes of PFT parameters in male and female mice from development through maturity. This study is also the first to investigate the influence of sex on baseline mouse pulmonary function during the period of greatest lung maturation.

Our measurements of murine lung function appear to take on a sigmoidal trend of growth during the preadolescent and pubertal phases of development, plateauing during adulthood. In humans, there is a relative paucity of data on age-related changes in lung function acquired via invasive measurement in healthy subjects, with the bulk of studies performed by noninvasive spirometric evaluation of dynamic flow rates such as the forced expiratory volume in 1 s (FEV1) and forced vital capacity (37–40). From what is known about age-dependent changes in FEV1 and lung capacities across the human lifespan, a sigmoidal trend is also seen in pulmonary function that parallels anthropomorphic growth in early life and reaches near steady-state mature values at ∼20–25 yr of age (35, 41). According to our data for inspiratory capacity, compliance, and hysteresis, rapid changes in mouse lung function during adolescence correspond to the period of greatest weight gain. In addition, the beginning of the lung function plateau appears to occur at a “young adult” age of 13 wk in mice, similar to humans. This plateau phase that we observe in mice correlates to the period of stability in human adult pulmonary function, seen at ∼20 to 35 yr of age (35). The decline in human lung function in older age is not yet apparent in mice by 23 wk of age except in the case of hysteresis, which is best explained by the peak and subsequent decline in surfactant-producing type 2 alveolar epithelial cells at 12 wk of age in mice (42). Thus, age and body size heavily influence pulmonary function in both species, and in humans forms the basis of pulmonary function scoring systems most commonly referencing these measurements to subject size and age (40, 41). We observed that maximal lung function was achieved relatively later in male mice (18–23 wk) than in females (∼13 wk), a phenomenon also described in humans (35). Of note, and in parallel to human data, compliance increased over time in our data set (43).

Allometric analysis has been previously applied to the lung size and cellular characteristics (32), however, this appears to be the first allometric analysis of lung function in male and female mice. Importantly, allometric scaling revealed that the changes in function of the lung largely scaled isometrically (i.e., the ratio of measured parameters with respect to allometric scaled parameters ∼1); hence, inspiratory capacity, lung compliance, and hysteresis were weight-dependent rather than sex-dependent. Thus, although human females have been attributed an inspiratory mechanical advantage conducive to increased lung compliance and inspiratory capacity due to a difference in thoracic dimensions (44), this sex-based difference in lung mechanics does not appear to carry over to mice. Interestingly, although a study found increased alveolar numbers and weight adjusted gas exchange area in female as compared with male mice, lung volume per se was not significantly different after adjustment for weight, similar to our results (45). The same study also found increased alveolar numbers of smaller size in female mice, a finding partly mirrored in the increased tissue damping in our lung function readouts and the slightly (but not significantly) smaller chord length in female mice in our study. In summary, our data suggest that differences in female and male mouse mechanical pulmonary function can be accounted for mostly by differences in body mass, which has implications for future research. For example, in the bleomycin model of pulmonary fibrosis, current guidelines support the use of both female and male mice for improved generalizability (2). However, the exact dosage of bleomycin to induce fibrosis has been unclear when comparing female and male mice. Our data support the use of a weight-adjusted amount of bleomycin independent of sex, as weight corresponds well to readouts for lung volume and seems to best explain the observed differences. Furthermore, our data support pooling pulmonary function data from male and female mice when sex as a biological variable has been shown to have no effect on respective disease models. Regardless, a separate analysis of male and female mice will be useful, as there will be less variation and no need for weight adjustment. This is especially true in disease models, where weight loss is a commonly observed effect.

Although our measurements correlated well with weight and age (up to 23 wk) in healthy mice, there are some inherent problems with this correlation for disease models. For example, in the murine bleomycin model of pulmonary fibrosis, weight loss goes hand in hand with considerable differences in lung function (46) and similar results hold for other disease models. In line with these considerations, if caused by obesity, increased weight leads to decreased compliance and increased tissue dampening (47). Of note, even in our data, there were no significant changes in lung function at later times (e.g., 18–23 wk) although weight still increased slightly. Thus, while our results can be used as reference for healthy mice, weight alone may not be a good parameter if changes in body mass are expected in (and maybe caused by) a model. Although body length might be helpful, and might be less influenced by aforementioned parameters, there are issues with inter- and intra-observer variance and state of the mice [e.g., living, dead and possibly based on paralyzing agent use (48)] that are not present to the same extent in body weight measurements. Consequently, when we correlated lung volume with body length, mass, and thoracic diameter measured via caliper, body mass showed the best ability to explain changes in lung function, closely followed by length.

The impact of anatomic resistive elements, particularly at the tracheal level, was relatively small throughout our investigation. The size of the human and murine trachea increases following birth but reaches its adult diameter during childhood (49). With a relatively constant tracheal size, it is not surprising that dynamic compliance, which is influenced by airway size, showed a trend similar to static compliance. The overall contribution of measured tissue damping to PFT assessment was present early in life, namely 2–3 wk of age, and initially greater in females but minimal thereafter in both sexes. These changes may reflect an early stabilization of alveolar architecture and remodeling of elastic tissue within the lung (8). A limitation of our study is that we did not investigate mice less than 2 wk of age, as these posed additional technical problems and further studies are needed.

Another limitation of our study was the use of a single mouse genetic strain. Strain-specific differences in PFTs have been reported in mice, though only between 10 and 14 wk of age. We chose to focus our study on C57BL/6J mice due to their widespread use in models of human lung diseases monitored by PFT assessment such as asthma (50), pulmonary fibrosis (24), and COPD/emphysema (51). In addition, testing mice at ages beyond 23 wk would potentially reveal when lung function starts to decline in this species, as predicted by PFT data in aging humans and structural changes in later life observed by others (36). Yet, a recent report could not detect any significant decline in either lung capacity or static compliance in aging C57BL/6J mice up to 24 mo but saw a decrease in tissue dampening (42).

In conclusion, this study profiled PFTs in male and female C57BL/6J mice from 2 to 23 wk of age, providing reference values for age- and sex-dependent changes in mouse lung biomechanics during development and adulthood. The most rapid developmental changes in lung structure and function of male and female mice occurred between 3 (weaning) and 8 (mature) weeks of age. Male mice became noticeably heavier than female mice at or ∼5 wk of age. We found that possible sex-based differences in lung function measurements are more likely body mass-based. We expect these data will facilitate future lung disease research by filling a critical knowledge gap in our field.

## DATA AVAILABILITY

The raw data used to create Table 1 and other data used for figures are posted as an Excel spreadsheet at https://doi.org/10.6084/m9.figshare.24871812.

## SUPPLEMENTAL DATA

10.6084/m9.figshare.24871812 Supplemental Allometric Analysis and Supplemental Figs. S1 and S2: https://doi.org/10.6084/m9.figshare.24871812.

## GRANTS

The research reported here was supported by the Department of Veterans Affairs, Veterans Health Administration, VISN 1 Career Development Award (to E.P.M.), NIH R03 AG074063 (to E.P.M.), and Single Ventricle Research Fund of Additional Ventures (to J.D.H. and E.P.M.). E.P.M. is a Pepper Scholar with support from the Claude D. Pepper Older Americans Independence Center at Yale School of Medicine under Grant No. P30AG021342); Thomas Bärnthaler is a recipient of the Apart-Mint (ÖAW) and Schrödinger Fellowship (FWF).

## DISCLOSURES

No conflicts of interest, financial or otherwise, are declared by the authors.

## AUTHOR CONTRIBUTIONS

T.B. and E.P.M. conceived and designed research; T.B., A.B.R., N.G., L.S., and E.P.M. performed experiments; T.B., A.B.R., L.S., and E.P.M. analyzed data; T.B., A.B.R., L.S., C.S.D.C., J.D.H., and E.P.M. interpreted results of experiments; T.B. and E.P.M. prepared figures; T.B. drafted manuscript; T.B., A.B.R., S.E., L.S., C.S.D.C., J.D.H., and E.P.M. edited and revised manuscript; T.B., A.B.R., S.E., L.S., C.S.D.C., J.D.H., and E.P.M. approved final version of manuscript.

## References

[1] Matute-Bello G, Frevert CW, Martin TR. Animal models of acute lung injury. Am J Physiol Lung Cell Mol Physiol 295: L379–L399, 2008. doi:10.1152/ajplung.00010.2008. 18621912 PMC2536793

[2] Jenkins RG, Moore BB, Chambers RC, Eickelberg O, Königshoff M, Kolb M, Laurent GJ, Nanthakumar CB, Olman MA, Pardo A, Selman M, Sheppard D, Sime PJ, Tager AM, Tatler AL, Thannickal VJ, White ES; ATS Assembly on Respiratory Cell and Molecular Biology. An Official American Thoracic Society Workshop Report: use of animal models for the preclinical assessment of potential therapies for pulmonary fibrosis. Am J Respir Cell Mol Biol 56: 667–679, 2017. doi:10.1165/rcmb.2017-0096ST. 28459387 PMC5800895

[3] Bates JH, Irvin CG. Measuring lung function in mice: the phenotyping uncertainty principle. J Appl Physiol (1985) 94: 1297–1306, 2003. doi:10.1152/japplphysiol.00706.2002. 12626466

[4] Vanoirbeek JAJ, Rinaldi M, De Vooght V, Haenen S, Bobic S, Gayan-Ramirez G, Hoet PHM, Verbeken E, Decramer M, Nemery B, Janssens W. Noninvasive and invasive pulmonary function in mouse models of obstructive and restrictive respiratory diseases. Am J Respir Cell Mol Biol 42: 96–104, 2010. doi:10.1165/rcmb.2008-0487OC. 19346316

[5] Schelegle ES, Mansoor JK, Giri S. Pirfenidone attenuates bleomycin-induced changes in pulmonary functions in hamsters. Proc Soc Exp Biol Med 216: 392–397, 1997. doi:10.3181/00379727-216-44187. 9402144

[6] Wollin L, Maillet I, Quesniaux V, Holweg A, Ryffel B. Antifibrotic and anti-inflammatory activity of the tyrosine kinase inhibitor nintedanib in experimental models of lung fibrosis. J Pharmacol Exp Ther 349: 209–220, 2014. doi:10.1124/jpet.113.208223. 24556663

[7] Vlahos R, Bozinovski S. Recent advances in pre-clinical mouse models of COPD. Clin Sci (Lond) 126: 253–265, 2013. doi:10.1042/CS20130182. 24144354 PMC3878607

[8] Ranga V, Kleinerman J, Ip MP, Sorensen J. Age-related changes in elastic fibers and elastin of lung. Am Rev Respir Dis 119: 369–376, 1979. doi:10.1164/arrd.1979.119.3.369. 375785

[9] Devos FC, Maaske A, Robichaud A, Pollaris L, Seys S, Lopez CA, Verbeken E, Tenbusch M, Lories R, Nemery B, Hoet PH, Vanoirbeek JA. Forced expiration measurements in mouse models of obstructive and restrictive lung diseases. Respir Res 18: 123, 2017. doi:10.1186/s12931-017-0610-1. 28629359 PMC5477381

[10] Bozanich EM, Collins RA, Thamrin C, Hantos Z, Sly PD, Turner DJ. Developmental changes in airway and tissue mechanics in mice. J Appl Physiol (1985) 99: 108–113, 2005. doi:10.1152/japplphysiol.01111.2004. 15817717

[11] Zucker I, Prendergast BJ. Sex differences in pharmacokinetics predict adverse drug reactions in women. Biol Sex Differ 11: 32, 2020. doi:10.1186/s13293-020-00308-5. 32503637 PMC7275616

[12] Regitz-Zagrosek V. Sex and gender differences in health. Science & Society Series on Sex and Science. EMBO Rep 13: 596–603, 2012. doi:10.1038/embor.2012.87. 22699937 PMC3388783

[13] Lam CSP, Arnott C, Beale AL, Chandramouli C, Hilfiker-Kleiner D, Kaye DM, Ky B, Santema BT, Sliwa K, Voors AA. Sex differences in heart failure. Eur Heart J 40: 3859–3868c, 2019. doi:10.1093/eurheartj/ehz835. 31800034

[14] LoMauro A, Aliverti A. Sex differences in respiratory function. Breathe (Sheff) 14: 131–140, 2018. doi:10.1183/20734735.000318. 29875832 PMC5980468

[15] Nguyen L, Castro O, De Dios R, Sandoval J, McKenna S, Wright CJ. Sex-differences in LPS-induced neonatal lung injury. Sci Rep 9: 8514, 2019. doi:10.1038/s41598-019-44955-0. 31186497 PMC6560218

[16] Card JW, Carey MA, Bradbury JA, DeGraff LM, Morgan DL, Moorman MP, Flake GP, Zeldin DC. Gender differences in murine airway responsiveness and lipopolysaccharide-induced inflammation. J Immunol 177: 621–630, 2006. doi:10.4049/jimmunol.177.1.621. 16785560 PMC2262913

[17] Tam A, Bates JHT, Churg A, Wright JL, Man SFP, Sin DD. Sex-related differences in pulmonary function following 6 months of cigarette exposure: implications for sexual dimorphism in mild COPD. PLoS One 11: e0164835, 2016. doi:10.1371/journal.pone.0164835. 27788167 PMC5082824

[18] Schulz H, Johner C, Eder G, Ziesenis A, Reitmeier P, Heyder J, Balling R. Respiratory mechanics in mice: strain and sex specific differences. Acta Physiol Scand 174: 367–375, 2002. doi:10.1046/j.1365-201x.2002.00955.x. 11942924

[19] Reinhard C, Eder G, Fuchs H, Ziesenis A, Heyder J, Schulz H. Inbred strain variation in lung function. Mamm Genome 13: 429–437, 2002. doi:10.1007/s00335-002-3005-6. 12226708

[20] Dutta S, Sengupta P. Men and mice: relating their ages. Life Sci 152: 244–248, 2016. doi:10.1016/j.lfs.2015.10.025. 26596563

[21] Geifman N, Rubin E. The mouse age phenome knowledgebase and disease-specific inter-species age mapping. PLoS One 8: e81114, 2013. doi:10.1371/journal.pone.0081114. 24312529 PMC3849212

[22] Fedorov A, Beichel R, Kalpathy-Cramer J, Finet J, Fillion-Robin J-C, Pujol S, Bauer C, Jennings D, Fennessy F, Sonka M, Buatti J, Aylward S, Miller JV, Pieper S, Kikinis R. 3D Slicer as an image computing platform for the quantitative imaging network. Magn Reson Imaging 30: 1323–1341, 2012. doi:10.1016/j.mri.2012.05.001. 22770690 PMC3466397

[23] Polosukhin VV, Degryse AL, Newcomb DC, Jones BR, Ware LB, Lee JW, Loyd JE, Blackwell TS, Lawson WE. Intratracheal bleomycin causes airway remodeling and airflow obstruction in mice. Exp Lung Res 38: 135–146, 2012. doi:10.3109/01902148.2012.658595. 22394287 PMC4046254

[24] Lawson WE, Cheng D-S, Degryse AL, Tanjore H, Polosukhin VV, Xu XC, Newcomb DC, Jones BR, Roldan J, Lane KB, Morrisey EE, Beers MF, Yull FE, Blackwell TS. Endoplasmic reticulum stress enhances fibrotic remodeling in the lungs. Proc Natl Acad Sci USA 108: 10562–10567, 2011. doi:10.1073/pnas.1107559108. 21670280 PMC3127925

[25] Salazar E, Knowles JH. An analysis of pressure-volume characteristics of the lungs. J Appl Physiol 19: 97–104, 1964. doi:10.1152/jappl.1964.19.1.97. 14104296

[26] Schroter RC. Quantitative comparisons of mammalian lung pressure volume curves. Respir Physiol 42: 101–107, 1980. doi:10.1016/0034-5687(80)90107-3. 6784203

[27] Venegas JG, Harris RS, Simon BA. A comprehensive equation for the pulmonary pressure-volume curve. J Appl Physiol (1985) 84: 389–395, 1998. doi:10.1152/jappl.1998.84.1.389. 9451661

[28] Boucher M, Henry C, Khadangi F, Dufour-Mailhot A, Tremblay-Pitre S, Fereydoonzad L, Brunet D, Robichaud A, Bossé Y. Effects of airway smooth muscle contraction and inflammation on lung tissue compliance. Am J Physiol Lung Cell Mol Physiol 322: L294–L304, 2022. doi:10.1152/ajplung.00384.2021. 34936511

[29] Sly PD, Collins RA. Chapter 7—Applied clinical respiratory physiology. In: Pediatric Respiratory Medicine (2nd ed.), edited by Taussig LM, Landau LI. Philadelphia: Mosby, 2008, p. 73–88.

[30] Tepper JS, Costa DL. Chapter 17—Methods, measurements, and interpretation of animal lung function in health and disease. In: Comparative Biology of the Normal Lung (2nd ed.), edited by Parent RA. San Diego: Academic Press, 2015, p. 305–351.

[31] Crowley G, Kwon S, Caraher EJ, Haider SH, Lam R, Batra P, Melles D, Liu M, Nolan A. Quantitative lung morphology: semi-automated measurement of mean linear intercept. BMC Pulm Med 19: 206, 2019. doi:10.1186/s12890-019-0915-6. 31706309 PMC6842138

[32] Stone KC, Mercer RR, Gehr P, Stockstill B, Crapo JD. Allometric relationships of cell numbers and size in the mammalian lung. Am J Respir Cell Mol Biol 6: 235–243, 1992. doi:10.1165/ajrcmb/6.2.235. 1540387

[33] Martin RD, Genoud M, Hemelrijk CK. Problems of allometric scaling analysis: examples from mammalian reproductive biology. J Exp Biol 208: 1731–1747, 2005. doi:10.1242/jeb.01566. 15855404

[34] Mitchell G, Skinner JD. An allometric analysis of the giraffe cardiovascular system. Comp Biochem Physiol A Mol Integr Physiol 154: 523–529, 2009. doi:10.1016/j.cbpa.2009.08.013. 19720152

[35] Sharma G, Goodwin J. Effect of aging on respiratory system physiology and immunology. Clin Interv Aging 1: 253–260, 2006. doi:10.2147/ciia.2006.1.3.253. 18046878 PMC2695176

[36] Pozarska A, Rodríguez-Castillo JA, Surate Solaligue DE, Ntokou A, Rath P, Mižíková I, Madurga A, Mayer K, Vadász I, Herold S, Ahlbrecht K, Seeger W, Morty RE. Stereological monitoring of mouse lung alveolarization from the early postnatal period to adulthood. Am J Physiol Lung Cell Mol Physiol 312: L882–L895, 2017. doi:10.1152/ajplung.00492.2016. 28314804

[37] Piccioni P, Tassinari R, Carosso A, Carena C, Bugiani M, Bono R. Lung function changes from childhood to adolescence: a seven-year follow-up study. BMC Pulm Med 15: 31, 2015. doi:10.1186/s12890-015-0028-9. 25885675 PMC4392458

[38] Karmaus W, Mukherjee N, Janjanam VD, Chen S, Zhang H, Roberts G, Kurukulaaratchy RJ, Arshad H. Distinctive lung function trajectories from age 10 to 26 years in men and women and associated early life risk factors—a birth cohort study. Respir Res 20: 98, 2019. doi:10.1186/s12931-019-1068-0. 31118050 PMC6532227

[39] Melén E, Guerra S. Recent advances in understanding lung function development. F1000Res 6: 726, 2017. doi:10.12688/f1000research.11185.1. 28620467 PMC5461903

[40] McGeachie MJ, Yates KP, Zhou X, Guo F, Sternberg AL, Van Natta ML, , et al Patterns of growth and decline in lung function in persistent childhood asthma. N Engl J Med 374: 1842–1852, 2016. doi:10.1056/NEJMoa1513737. 27168434 PMC5032024

[41] Talaminos Barroso A, Márquez Martín E, Roa Romero LM, Ortega Ruiz F. Factors affecting lung function: a review of the literature. Arch Bronconeumol (Engl Ed) 54: 327–332, 2018. doi:10.1016/j.arbr.2018.04.003. 29496283

[42] Schulte H, Muhlfeld C, Brandenberger C. Age-related structural and functional changes in the mouse lung. Front Physiol 10: 1466, 2019. doi:10.3389/fphys.2019.01466. 31866873 PMC6904284

[43] Huang J, Zhang H, Zhang M, Zhang X, Wang L. Reference values for resistance and compliance based on the single occlusion technique in healthy infants from Southeast China. J Thorac Dis 8: 513–9, 2016. doi:10.21037/jtd.2016.02.69. 27076948 PMC4805831

[44] Bellemare F, Jeanneret A, Couture J. Sex differences in thoracic dimensions and configuration. Am J Respir Crit Care Med 168: 305–312, 2003. doi:10.1164/rccm.200208-876OC. 12773331

[45] Massaro GD, Mortola JP, Massaro D. Sexual dimorphism in the architecture of the lung's gas-exchange region. Proc Natl Acad Sci USA 92: 1105–1107, 1995. doi:10.1073/pnas.92.4.1105. 7862643 PMC42646

[46] Bärnthaler T, Theiler A, Zabini D, Trautmann S, Stacher-Priehse E, Lanz I, Klepetko W, Sinn K, Flick H, Scheidl S, Thomas D, Olschewski H, Kwapiszewska G, Schuligoi R, Heinemann A. Inhibiting eicosanoid degradation exerts antifibrotic effects in a pulmonary fibrosis mouse model and human tissue. J Allergy Clin Immunol 145: 818–833.e11, 2020. doi:10.1016/j.jaci.2019.11.032. 31812575

[47] Lu FL, Johnston RA, Flynt L, Theman TA, Terry RD, Schwartzman IN, Lee A, Shore SA. Increased pulmonary responses to acute ozone exposure in obese db/db mice. Am J Physiol Lung Cell Mol Physiol 290: L856–L865, 2006. doi:10.1152/ajplung.00386.2005. 16373670

[48] Stephens RB, Karau KH, Yahnke CJ, Wendt SR, Rowe RJ. Dead mice can grow—variation of standard external mammal measurements from live and three postmortem body states. JMAMMAL 96: 185–193, 2015. doi:10.1093/jmammal/gyu022.

[49] Furlow PW, Mathisen DJ. Surgical anatomy of the trachea. Ann Cardiothorac Surg 7: 255–260, 2018. doi:10.21037/acs.2018.03.01. 29707503 PMC5900092

[50] Debeuf N, Haspeslagh E, van Helden M, Hammad H, Lambrecht BN. Mouse models of asthma. Curr Protoc Mouse Biol 6: 169–184, 2016. doi:10.1002/cpmo.4. 27248433

[51] Hong Y, Kim Y-S, Hong S-H, Oh Y-M. Therapeutic effects of adipose-derived stem cells pretreated with pioglitazone in an emphysema mouse model. Exp Mol Med 48: e266, 2016. doi:10.1038/emm.2016.93. 27765950 PMC5099424

